# The Dental Section of the International Medical Congress

**Published:** 1885-03

**Authors:** 


### Editorial.

### THE DENTAL SECTION OF THE INTERNATIONAL MEDICAL CONGRESS.

Judging from the communications received relative to the editorial in the February number of this journal, the dentists are thoroughly aroused to the necessity for a proper organization of the profession, that it may make a creditable exhibition at Washington in 1887. That article was written for the purpose of awakening an interest in what should prove a memorable event in our professional history. We were aware that action was already contemplated, and steps had even been taken to open the work. The Chairman of the section of Oral Surgery in the American Medical Association had been at work, and the Chicago Dental Society had appointed a committee to further the object which all of us should have at heart. That committee has issued a circular, calling attention to the necessity for some concert of action on the part of the dentists.

Just too late for insertion in the February number came the following letter from the Secretary General, which gives official assurance that the section has been decided upon.

INTERNATIONAL MEDICAL CONGRESS,
WASHINGTON, 1887.

OFFICE OF THE SECRETARY GENERAL,
WASHINGTON, D. C., Feb. 1, 1885.

DEAR SIR:

I have the honor to inform you that it has been decided by the General Committee of the International Congress to have a Section of Dental and Oral Surgery. The officers of the section have not yet been selected. Very respectfully yours,

JOHN S. BILLINGS,
Secretary General.

No official information as to the choice of officers (if any of them have been selected) has yet been made public, but at the late dinner of the Odontological Society in New York, it was intimated by one who should be in a position to know, that Prof. Taft, of Cincinnati, has been named by the committee as Chairman of the section. This choice, if it shall be confirmed, will probably be as satisfactory to the dentists as any that could be made. Prof. Taft has a world-wide fame as an author and teacher; he has always been prominently connected with dental societies, and his selection would doubtless meet with as little opposition as would that of any man. It is essential that some one should be chosen who can bring to his support an undivided profession, and Prof. Taft would probably obtain as cordial co-operation as any one who could be named. Concerning his abilities as a presiding officer, and his talent as an organizer, we have no personal knowledge, but he certainly is not without experience. Whoever shall be chosen by the General Committee of the Congress, he must be heartily sustained by the dentists if we would see the section made successful.

The Congress of 1881, meeting as it did in London, the home of so many eminent dentists, a city distinguished for the hospitality of its professional men, was notably successful in its social aspects. There must be no stint in the entertainments offered to the guests of American dentists in 1887. We cannot afford to be outdone by our English brethren, and although the city of Washington does not enjoy the advantage of such a large body of local dentists as has London, the whole profession of America should join in extending a welcome, and in proffering such social attentions to the foreign members of the section as shall be worthy them and ourselves. To this end due organization of the whole profession is necessary, and the various State and National Societies should take the matter in hand at an early day.

Some provision must be determined upon for representation in the section of those who are without the distinguishing medical degree that the General Committee has determined upon as requisite for membership in the general Congress. They must be admitted with all the privileges of the section, for a Dental Congress composed exclusively of medical men would be a failure from the start. A number of plans have been proposed, any of which might reach the desired end, but the final decision must of necessity rest with the General Committee, and with the officers of the section when chosen. In the meantime a careful and temperate consideration of the whole question by the profession will greatly aid in its proper settlement.

